# No evidence for spatial variation in predation risk following restricted-area fox culling

**DOI:** 10.1186/s12898-019-0235-y

**Published:** 2019-04-25

**Authors:** Jim-Lino Kämmerle, Sarah Niekrenz, Ilse Storch

**Affiliations:** 1 Chair of Wildlife Ecology and Management, University of Freiburg, Freiburg, Germany; 2 Forest Research Institute of Baden-Württemberg FVA, Freiburg, Germany

**Keywords:** Artificial nest, Density, GAM, Hunting, Mesopredator, Predator control, Wildlife camera trap

## Abstract

**Background:**

Predation and predator abundance may significantly affect bird populations, especially ground nesting species, because nest predation is often the major cause of nest failure. Predator control by means of culling is frequently employed to benefit threatened prey species or to increase the abundance of small game species for hunting. The red fox (*Vulpes vulpes*), a generalist mesopredator of global relevance, is a major target of predator control. Commonly, in central Europe, red fox culling efforts intended to benefit prey species remain restricted to small areas. It is unclear, however, whether such restricted-area culling effectively lowers predation risk at a site or whether red fox abundance is more important than culling in shaping predation risk. We conducted an experiment using 273 camera supervised artificial nests at multiple study sites in clusters of hunting concessions with or without targeted fox culling in a fragmented montane forest landscape in Germany.

**Results:**

Using generalized additive models, we assessed whether incentivized recreational culling of red foxes was associated with local reductions in an index of predation risk and fox occurrence probability, or whether both were explained by red fox abundance instead. Final models indicated that restricted-area culling of red foxes was not associated with local reductions in predation risk, nor lower probability of a fox sighting, even for the plots with the largest hunting bags. Predation risk at a plot instead appeared to be driven by variation in the abundance of red foxes in the landscape surrounding the plots. After accounting for fox abundance, we found no additional relationship of artificial nest predation risk with landscape configuration.

**Conclusions:**

Our results imply that the scale and intensity of predator control achieved by incentivized recreational hunting was ineffective at altering fox abundance patterns and associated predation risk. We thus find no evidence to support incentives for uncoordinated recreational red fox culling as a conservation measure.

**Electronic supplementary material:**

The online version of this article (10.1186/s12898-019-0235-y) contains supplementary material, which is available to authorized users.

## Background

Profound landscape modifications and widespread extirpation of apex predators have changed the abundance and composition of predator communities in natural and semi-natural systems across the globe [1–4]. These ‘mesopredator release’ [5] ecosystems are often characterized by altered trophic interactions, including high mesopredator abundance and strong predation pressure on their prey [1, 6, 7]. Predation and predator abundance are particularly relevant for bird populations, because breeding success is a crucial determinant of their development [8–10] and nest predation is often the most frequent cause of nest failure [11–13]. Rates of nest predation increase and reproductive success of forest birds decreases in fragmented forest mosaic landscapes (nest predation [14–16]; reproductive success [17–19]). This may be partially attributable to a high abundance of generalist predators in mosaic landscapes [20–22], if generalist predators also frequently use all elements of the landscape matrix [19]. Owing to the difficulty of observing nest predation events in birds, a large amount of studies have used artificial nests as a measure of predation risk in relation to habitat and landscape characteristics (e.g. [16, 23–25]). A major shortcoming of most studies using artificial nests is—apart from their limited comparability with real nest-loss [24, 26]—that they were unable to identify predator species with certainty and that most studies could not directly link artificial nest loss to predator abundance, but only to habitat and landscape configuration. The use of camera traps to identify predators of artificial nests could alleviate the former issue, while independent measures of predator abundance collected at the study sites are required for the latter.

The red fox (*Vulpes vulpes*) is a mammalian generalist mesopredator with global relevance for conservation and wildlife management [27]. In central European forest-farmland mosaic landscapes red foxes regularly utilize all land cover types [28–30], thus linking high predator abundance in such landscapes with an elevated predation risk for species inhabiting forest fragments. Even though generalist predators may rarely target nests of forest birds specifically [16, 31], predation rates may nonetheless be high in fragmented landscapes because of the elevated encounter risk in smaller habitat patches [19, 32].

Predator control by means of culling is often employed in wildlife management with the goal of benefitting threatened prey species or to increase the abundance of game species for hunting [7, 33]. Predator control can benefit a variety of prey taxa [9, 34, 35], including birds such as grouse [36, 37], and often has clear effects on the reproductive parameters of the target species [9, 38]. Effects on prey species are, however, often limited to programs that effectively reduce predator abundance [35]. Although generalisation is difficult owing to the multitude of affected species worldwide, mesopredator control programs typically only have short-term impacts on predator populations [7].

In practice, the realized culling intensity is often limited by practical constraints such as available person-hours or the limits imposed by hunting legislation, particularly when recreational hunting is the main means of predator control. A major hurdle for control programs using recreational hunting in Central Europe is the variation in commitment among individual hunters [39]. This often leads to spatially structured harvests from continuously distributed predator populations [7], thus creating localized source-sink dynamics in the landscape with unclear effects on the abundance of common species such as red foxes. Whether such uncoordinated restricted-area culling (i.e. targeted fox culls in individual hunting concessions) reduces red fox abundance is controversial and effects are likely temporary [40–43]. Furthermore, predator control aiming at conservation of a threatened prey species ultimately does not target a reduction in predator abundance, but a decrease in predation rates. Numerous studies have addressed culling effects on red fox abundance (e.g. [40, 44, 45]) and local benefits for prey populations (e.g. [46–48]) in different systems. Little is known, however, on how spatial heterogeneity in culling intensity (e.g. in the form of incentivized recreational hunting) affects variation in predation risk across the landscape.

We conducted an experiment using camera supervised artificial nests at multiple sites in fragmented montane forest landscapes covering an intensity gradient of incentivized recreational restricted-area culling of red foxes. We assessed whether restricted-area culling of foxes was associated (1) with local reduction in an index of predation risk and (2) with local reductions in fox occurrence probability, or whether red fox abundance in the surrounding landscape was more important than culling in shaping predation risk.

## Methods

### Study area

**Fig. 1:**
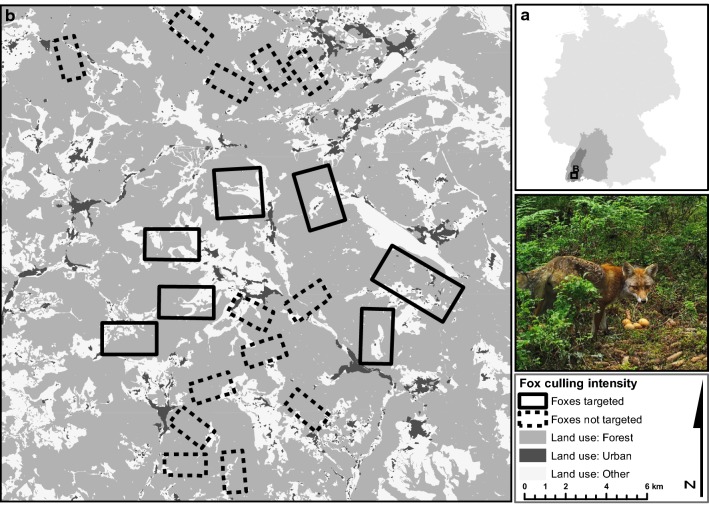
Overview of the study sites for the artificial nest experiments (**b**) in the southern Black Forest mountain range in southwestern Germany (**a**). The insert image shows a typical artificial nest situation at the plots during a predation event

We conducted our study in the southern Black Forest mountain range in South-Western Germany (max. elevation 1493 m asl; Fig. 1). The area is characterized by a land use mosaic dominated by forests (approximately two-thirds forest, [49]). Forests are fragmented by settlements, single farms and livestock pastures (Fig. 1). In this study, we focused on medium to high elevation montane forests at an altitude of 800 to 1400 meters above sea level. Forest communities in our study area were predominately mixed stands of *Fagus sylvatica*, *Abies alba* and *Picea abies*. Management of red fox populations is incentivized in the study area for conservation purposes, because red foxes are considered important predators of capercaillie (*Tetrao urogallus*), a locally threatened prey species [50].

Shooting and hunting of game in the study area is organized concession-based according to German legislation as a mixture of privately allotted hunting concessions and hunting grounds managed by the state, both ranging between approximately 100 to 1.500 ha in size. Culling of red foxes and other mammalian mesopredators (e.g. *Martes* spp., *Meles meles*) is permitted without quota during the hunting season. Accordingly, the culling intensity in each concession is mainly determined by the commitment of the individual hunters, creating a mosaic of varying hunting intensity across the landscape. The majority of red fox culling in our study area was conducted by shooting during the winter months (December–February) at baited sites with snow cover. In this study, we selected hunting concessions based on their hunting effort with regards to red foxes (i.e. no hunting, occasional shooting, targeted removal). We interviewed concession holders to establish their hunting strategy with regards to foxes and the average size of red fox shooting bags in former years. Incentives to cull foxes were provided in our study through local chapters of the state hunters association aiming at capercaillie conservation (state law requires conservation imperative to permit certain types of fox hunting), annual fox removal events organized by local chapters of the hunters association (1 week each) and encouragement by the state hunters association to sell the fur through provision of infrastructure and awards for high shooting bags. Within the set of hunting concessions, we selected a total of 20 study sites characterized by either targeted removal of red foxes or no fox removal (henceforth high-hunting and low-hunting ‘study sites’; B in Fig. 1). Study sites were located within sets of several spatially adjoining groups of hunting concessions with similar culling strategy. We chose this approach to ensure that study sites where fully contained by areas of comparable culling strategy. We selected study sites with high and low hunting in areas of similar landscape composition and similar predicted red fox abundance [51], to minimize potential bias of selecting areas with high hunting bags in areas of high fox abundance. We finally selected a study area of approximately 30 × 60 km, comprising 20 rectangular study sites within 26 hunting concessions (hunting concessions ranged in size between 140 and > 1.000 ha; mean = 700 ha, SD = 400). The total surveyed concession area amounted to approximately 18.000 ha. Hunting bags ranged from zero to five foxes 100 ha^−1^ shot annually across the study area. Red fox densities in the area are unknown, but home-range sizes obtained by VHF telemetry in a low valley of the study area [28] suggest moderate to intermediate density [52].

### Survey design

We assigned plots to the study sites using a systematic grid of 500 m spacing. A maximum effort of 140 plots per field season was distributed across the 20 study sites. Clusters of six plots each were assigned to 13 low-hunting study sites and 10–12 plots each in seven high-hunting study sites. The final selection of plots from the grid within the study sites was stratified by the expected abundance of foxes in the area using a model of fox abundance based on landscape composition and environmental productivity (using predictions as in Güthlin et al. [21]). We conducted the artificial nest experiments for two consecutive years in 2017 and 2018: each year between the end of May and the end of July in two consecutive sessions of 21 days (i.e. to coincide with the reproductive period of capercaillie, the main focal species of conservation concern in the area). Each session included approximately 70 plots.

We used camera supervised artificial nests as an index for variation in predation risk across the study area, but made no attempt to infer real nest loss of capercaillie based on artificial nests. We placed motion-triggered infra-red (IR) flash automatic wildlife cameras (Bushnell Trophy Cam HD Aggressor Low Glow) at the previously assigned plots, locating each plot using standard handheld GPS devices. Camera installment protocol, camera sensitivity and trigger settings were held constant across all plots to standardize detection probability. All field staff wore rubber boots when traversing from the vehicle to the plot location (boots were kept in boxes containing forest soil for the remainder of the time) to minimize bias introduced by scent trails. We placed four brown chicken eggs (medium size) on the ground at a distance of approximately one to two meters in front of the cameras (Fig. 1) in a small depression using a new pair of single-use rubber gloves at each plot. We made no attempt to otherwise mimic a natural nest situation. Chicken eggs in each year and session originated from the same egg farm and had the same delivery date for each complete study session to minimize bias due to differences in egg smell among nests. We recorded a number of plot scale variables at each plot location. We measured vertical nest cover (i.e. from the side) as the average of all four cardinal directions estimated from 10 m distance using a 50 × 50 cm checker board held on ground level directly in front of the nest. We estimated horizontal nest cover (i.e. from above) by standing on the plot location before placing the eggs and estimating the vegetation cover within 1 m^2^ above the nest at 1 m, 3 m and crown height. Finally, we estimated the proportion of ground covered by vegetation and coarse woody debris that could resist red fox movement in a 20 m radius around the plot location as well as the distance to the next forest edge and the type of edge (i.e. forest-clearing, forest–forest for two stand types, forest-pasture). We noted how many eggs had been taken when retrieving the cameras after 3 weeks.

## Data analysis

### Data preparation

We retrieved all pictures from the cameras and determined the periods over which they had been operational. We limited the study period to the first 21 days for all plots and removed plots where cameras malfunctioned. All pictures were sorted to species level. Due to the use of IR-flash we were unable to distinguish with certainty between pine martens (*Martes martes*) and beech martens (*Martes foina*) and thus refrained from inferences about patterns of marten abundance. All further data handling and analysis were carried out within the R Statistical Environment version 3.5.0 [53]. We used package camtrapR version 0.99.9 [54] and the free software exiftools to extract metadata from pictures and group pictures into events. Image sequences more than 5 min apart were considered independent events based on visual inspection of the data. We prepared two response variables from the data: (a) whether the nest was predated or not regardless of the predator species (binary; ‘nest predation’) and whether a picture of a fox had been recorded at the nest (binary; ‘fox occurrence’; frequency distribution prohibited direct analysis of count data).

In addition to plot-based covariates, we prepared a number of environmental predictors to explain variation in predation risk and fox occurrence across the landscape based on our hypotheses. We quantified landscape heterogeneity using the Shannon Index [21, 55] with the proportions of the four land cover types in the study area (i.e. forest, pasture, arable, settlement) at the scale of a fox home-range in the area (i.e. 197 ha, radius ≈ 800 m; [28]). For each plot, we also calculated the distance to agricultural land use types and human settlements (including single farms), the proportion of human land use types and the proportion of forest cover in a 250 m buffer around plot locations (i.e. half the distance between plots) as well as the distance to the nearest forest edge (negative values inside the forest). We processed raw hunting bag data into a continuous predictor of culling intensity across the study area. We obtained governmental hunting bag data at the concession level for both years of the study (i.e. hunting season 2016/2017 and 2017/2018) and assigned red fox hunting bags for each study year to the centroid of each hunting concession in our study area. We repeated this for all surrounding hunting concessions up to a distance of well beyond one fox home-range diameter distance to the study area. We normalized the hunting bags by the concession area (i.e. foxes culled km^−2^). We then obtained a continuous predictor of hunting intensity (separately for each study year) by interpolating values using a two-dimensional minimum-curvature tension spline that exactly passes through the input points in software ArcMap 10.5.1 [56] to obtain a representative estimate of variation in hunting pressure across the landscape. The interpolated normalized bag data were extracted at each artificial nest location as predictor in the analysis. Finally, we estimated red fox abundance after the main culling period (i.e. January–March) for each plot using empirical data on red fox abundance collected at the study sites in each year between March and May (for a full description of the data collection protocol see [57]). We used the mean number of camera trap events of red foxes per week across all camera locations in the dataset described by [57] within 1.6 km distance to the artificial nest location (i.e. using the approximate diameter of an average fox home-range in the area: i.e. 197 ha, radius ≈ 800 m [28]) as an empirical estimate of variation in relative red fox abundance (henceforth: ‘fox abundance’) at the landscape scale.

### Statistical analysis

All analyses were performed within the R Statistical Environment (version 3.5.0; [53]). We assessed all environmental and plot-based covariates for collinearity by calculating pairwise Pearson correlations for each predictor in the set to avoid wrongful interpretation of collinear predictors in the model [58]. We considered all variable pairs as potentially collinear whose pairwise correlation coefficient was above a conservative threshold of |r| > 0.5 and performed pre-selection given our set of hypotheses.

We modelled the probability of an artificial nest being predated (1 = predated; 0 = not predated) and the probability of encountering foxes at the nest location (1 = fox detected; 0 = fox not detected) using generalized additive models (GAM) from package mgcv version 1.8–24 [59, 60] with a binary response and a logit link. We used cubic regression splines with shrinkage for continuous predictors, limiting the flexibility of the splines to a maximum of three degrees of freedom. We fitted full models for each hypothesis including all predictors at the plot and landscape scale and selected important predictors using shrinkage in the full model. The full model for nest predation included the proportion of forest around the plot, the distance to the forest edge, the distance to the closest human settlement, the fox hunting intensity, the empirical red fox abundance in the landscape around the plot, the vertical and horizontal vegetation cover at the nest site, the slope at the site, the percentage of ground covered by structures resisting fox movements and whether the nest was located within 100 m of a habitat edge as well as the year of study (factor: two levels) and the session of the experiment (factor: two levels). The full model for fox presence differed by inclusion of the Shannon index of land cover diversity rather than the proportion of forest, no inclusion of horizontal vegetation cover of the nest and the addition of a predictor specifying whether the nest had been predated or not (Factor: two levels). The level of significance was set at α = 0.05.

## Results

A total of 273 plots with artificial nests delivered usable data in 2017 (N = 130) and 2018 (N = 143). The proportion of predated nests was similar among study years (2017: 51/130 = 39.2%; 2018: 60/143 = 42.0%). Foxes caused a mean of 43.2% of the predated nests, with 41.2% (21 of 51) of predated nests in 2017 and 45% (26 of 60) in 2018. Martens caused the majority of the remaining predation events (2017: 39.2%; 2018: 38.3%). Few events were caused by corvid birds, mainly jays (*Garrulus glandarius*), and wild boar (*Sus scrofa*); some remained unknown (2017: 1.5%; 2018: 8.2%).

### Model results

**Fig. 2:**
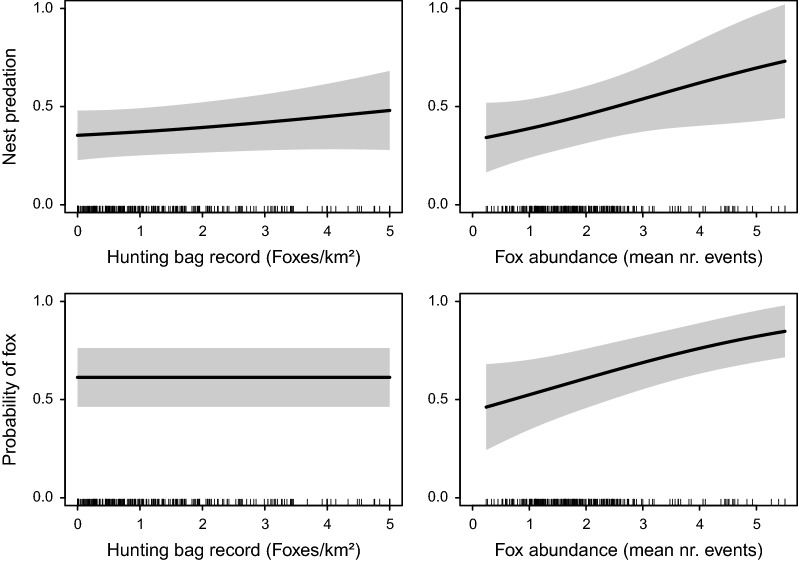
Conditional effect plots for the probability of an artificial nest being predated (top row) and the probability of a fox occurrence at the artificial nest (bottom row) as a function of the hunting bag record at a plot (as red foxes culled km^−2^) and the relative fox abundance in the landscape surrounding the plot (as mean number of red fox events at cameras within one home-range diameter distance to the plot). All other variables were set to the mean

In the model of predation risk, the probability of an artificial nest being predated increased significantly with increasing red fox abundance in the surrounding landscape (Table 1, see also Fig. 2). The relationship between nest predation and culling intensity had a small positive slope, but was non-significant. No plot- or landscape scale covariates were significant and most were shrunk to zero, except for slope, the distance to the forest edge and horizontal nest cover at 3 m height.

**Table 1:** GAM results for (a) predation of and (b) fox occurrence at artificial nests

(a) Model nest predation				
Predictors	Estimate	SE	z-value	p-value
Intercept	− 0.463	0.255	− 1.819	0.069
Edge-Yes	0.097	0.265	0.367	0.714
Year-2018	0.323	0.283	1.141	0.254
Session-2	− 0.273	0.259	− 1.055	0.291

Predictors	Edf	*χ* ^2^	p-value
Prop. Forest	< 0.01	0	0.549
Edge Dist.	0.481	1.013	0.131
Hum. Dist.	< 0.01	0	0.809
*Hunt. Bag*	*0.725*	*1.771*	*0.101*
*Fox Abund.*	*0.885*	*3.890*	*0.021*
%shrub	< 0.01	0	0.760
Slope	0.581	1.267	0.128
NcV	< 0.01	0	0.508
NcH_1m	< 0.01	0	0.349
NcH_3m	0.502	0.973	0.158
NcH_can	< 0.01	0	0.790

(b) Model fox occurrence				
Predictors	Estimate	SE	z-value	p-value
Intercept	− 1.047	0.296	− 3.542	< 0.001
Edge-Yes	0.047	0.276	0.171	0.864
Year-2018	0.558	0.303	1.843	0.065
Session-2	− 0.162	0.274	− 0.589	0.556
Pred.-Yes	1.514	0.270	5.600	< 0.001

Predictors	Edf	*χ* ^2^	p-value
Shannon	< 0.01	0	1.000
Edge Dist.	< 0.01	0	0.648
Hum. Dist.	< 0.01	0	0.893
*Hunt. Bag*	< *0.01*	*0*	*0.543*
*Fox Abund.*	*1.075*	*8.581*	*0.001*
%shrub	< 0.01	0	0.310
Slope	0.323	0.470	0.220
NcV	0.328	0.406	0.261

Edge-Yes: artificial nest within 100 m of forest habitat edge; Prop. Forest: proportion of land cover forest within 250 m around plot; Shannon: Shannon index of landscape heterogeneity; Edge Dist: Distance to forest edge; Hum. Dist: distance to closest settlement; Hunt. Bag: size of normalized hunting bag in area around plot (foxes/km^2^); Fox Abund: emprirical fox abundance in landscape around plot (mean nr. foxes); %shrub: percentage of ground covered by structures hindering fox movement; Nc: nest cover (vertical; horizontal at three levels: 1 m, 3 m and canopy level)

Parameter estimates, standard errors and p-values are provided for factor covariates (top section); estimated degrees of freedom and p-values (without considering uncertainty in smoothing parameter estimates) are provided for the smooth terms (bottom section). Predictors of Fig. 2 are highlighted italics

In the model of fox occurrence, the probability of a fox sighting at an artificial nest location likewise mainly increased with fox abundance in the surrounding landscape (Table 1). There was no effect of culling intensity on the probability of encountering foxes at the nest, but there was a significantly higher likelihood of a fox sighting if the artificial nest had been predated during the experimental period. Most remaining covariate effects were shrunk to zero, except for slope and vertical nest cover, which were both not significant. In both models, there was no significant difference between years or study sessions (Table 1).

## Discussion

Restricted-area culling of red foxes aiming for fox population control was not associated with locally reduced predation risk nor with a lower probability of a fox occurrence at a study site, even when hunting bags were comparatively large (i.e. when bag records exceeded the expected red fox population density at a site). This is in line with previous work on the effectiveness of local, isolated culls to control red fox abundance [41]. Instead, predation risk appeared to be driven mainly by variation in the abundance of red foxes in the landscape surrounding the plots.

In foxes and other mammalian mesopredators there is a negative relationship between population density and home-range size [52]. Based on this relationship and estimated fox home-range sizes in the study area [28], red fox density at the study sites may be assumed to be (well) below five individuals km^−2^. Accordingly, hunting bag sizes in concessions targeting foxes during our study were equal to or higher than the assumed fox density in the area. Fox removal of this order of magnitude has been found to be associated with local suppression of fox abundance relative to the carrying capacity [43]. Reports from hunters in our study area indicate, however, that sex ratios in the culled foxes are extremely male-biased by as much as five to one animals culled (unpublished data), thus indicating that predator control may have been ineffective at creating local reductions in predation risk because mainly transient male animals were culled. Although we found no indication that restricted-area culling led to local variation in fox abundance, previous work has shown that networks of estates with fox control can suppress regional fox abundance to a certain degree [42], but the extent of such effects in our study area is unclear. Given the potentially severely biased sex ratios of culled animals in the study area, effects on the reproducing population appear unlikely. In addition, previous studies that modelled effects of restricted-area culling on fox populations using population models concluded that immigration from surrounding areas is a key process in determining the effects of culling on local fox abundance [43, 61, 62]. In the case of our study area, concessions practicing predator control were imbedded in a mosaic of concessions without fox control (Fig. 1), thus providing potential sources of fox individuals to compensate the cull. Cull sites in our study area are also easily reachable for foxes from the wider landscape given the potential dispersal distances of red foxes [63]. This suggests that, although restricted-area culls might have created localized sinks, the introduced mortality was not sufficient to affect source populations in the wider landscape.

Predation rates on artificial nests as a proxy for nest predation risk have repeatedly been related to landscape configuration, including edge effects in forest farmland ecotones [14, 64], landscape-scale edge effects within the forest matrix [16] and effects of landscape heterogeneity [23, 65]. Higher predation rates in farmland-forest mosaic landscapes and small forest patches are likely attributable to a high abundance of generalist predators in these landscape types [20–22] together with a higher likelihood of encountering a predator in small forest habitat patches [19, 32]. We did not find additional effects of landscape composition on artificial nest predation risk in our study after accounting for red fox abundance. In contrast to the majority of previous studies, which were unable to link predation rates directly to predator abundance, we show that predation rates of artificial nests were directly related to the abundance of red foxes in the landscape surrounding a site.

### Technical considerations

We used interpolated normalized hunting bags (i.e. foxes culled km^−2^) as a proxy for actual culling intensity in our study areas. Hunting bag data are, however, not corrected for the effort spent hunting and there is the risk of high hunting bags reflecting high fox abundance in an area (i.e. that with equal effort more foxes are culled in areas of high density) instead of differences in culling intensity. The hunting concessions used in this study were selected based on their culling strategy (i.e. targeted fox removal vs. no fox removal) rather than based on bag data and were placed within areas of comparable expected red fox abundance (range of expected relative fox abundance: ‘high’ sites: 1.25–2.58; ‘low’ sites: 1.11–2.48; whole study area: 1.02–5.52). Accordingly, we are confident that the differences in hunting bags actually reflect differences in culling intensity within the study concessions. This is supported by the lack of correlation of normalized hunting bag size in the study area with our empirical measure of red fox abundance (i.e. |r| < 0.01), thus confirming our selection of study sites by culling strategy within similar landscape composition.

Although artificial nest experiments have often been used to study patterns in nest predation risk [16, 23, 25, 66], they are unsuitable for inferring rates of nest loss in ground nesting birds due to differences in predation rates between real and artificial nests [24, 26, 67]. This discrepancy in predation rates is probably due to differences in nest concealment and appearance, nest defence and different predator faunas [24, 26, 68, 69]. We did, however, not attempt to use artificial nests to infer patterns of real nest loss in this study. Instead, we used artificial nests as a standardized measure of predation risk that allowed for replication across the study area and excluded typical sources of heterogeneity in nest predation rates [16, 70, 71]. In addition, all efforts were taken to standardize the deployment protocol, appearance and smell of artificial nests. The use of such an index thus allowed us to infer patterns in predation risk associated to predator abundance and landscape composition as well as predator manipulation (i.e. culling) on a spatial scale that would be highly impractical to cover using real nests of ground nesting bird species in forests of Central Europe [16].

## Conclusions

In summary, restricted-area culling of red foxes was not associated with local reductions in predation risk, nor a lower probability of detecting foxes at a site during our study. Instead, predation risk reflected variation in the abundance of red foxes, the major nest predator, in the surrounding landscape. This emphasizes the importance of quantifying red fox abundance when evaluating the effectiveness of predator control. The scale and intensity of predator control achieved by incentivized recreational hunting, as practiced in the study area, thus appear insufficient to alter fox abundance patterns and the associated predation risk during the most relevant time for conservation (i.e. during the reproductive period of most ground nesting birds in the area). This suggest that, in order for predator control to be effective, larger areas with homogeneous culling regime are required and that removal intensities may need to be higher than the maximum values recorded in our study. Whether this can be achieved by means of recreational hunters and given the current hunting legislation in the state is, however, unclear. Accordingly, we currently find no evidence to support incentives for uncoordinated recreational red fox culling as a conservation measure, as commonly practiced in Central Europe.

## Additional file

**Additional file 1.** Dataset for the analysis performed in this article.
